# Antibiotic Use in Livestock: A Driver of Resistance in Africa and the Path to Safer Alternatives

**DOI:** 10.1002/mbo3.70122

**Published:** 2025-11-10

**Authors:** Mercy A. Alabi, Hafizah Y. Chenia, Johnson Lin

**Affiliations:** ^1^ Discipline: Microbiology, School of Life Sciences, College of Agriculture, Engineering and Science University of KwaZulu‐Natal Durban South Africa

**Keywords:** animal feed additive, animal growth promoters, antibiotic resistance, antibiotic resistance transfer, antibiotic stewardship

## Abstract

Antibiotics are widely used in animal production for disease treatment, prevention, and as growth promoters at subtherapeutic doses. Across Africa, various antibiotic classes, including beta‐lactams, macrolides, tetracyclines, phenicols, fluoroquinolones, aminoglycosides, and polymyxins, have been incorporated into animal feed to enhance growth and productivity. However, the continuous supplementation of animal feed with antibiotics exerts selective pressure on bacteria, accelerating the development of antibiotic resistance. This misuse in animal agriculture significantly contributes to the growing global threat of antibiotic resistance, affecting animal, human, and environmental health. Resistant strains of *Staphylococcus aureus, Pseudomonas aeruginosa, Escherichia coli, Klebsiella pneumoniae*, and *Salmonella* species have been widely reported in animal, human, and environmental samples. The transmission of antibiotic‐resistant bacteria from animals to humans can occur through direct contact. It can also result from exposure to contaminated manure, wastewater, or consumption of contaminated animal products. This poses a major public health challenge in Africa. To mitigate antibiotic resistance, the use of antibiotics as growth promoters in animal farming must be restricted. Alternative feed additives such as probiotics, prebiotics, and phytobiotics have shown potential as sustainable replacements. Educating farmers on antibiotic risks and sustainable alternatives is crucial. Furthermore, governments must implement strict regulations to control the sale and misuse of antibiotics in livestock production. The review aims to present the harm of antibiotic misuse in livestock farming and emphasize the need for alternative growth promoters, ultimately reducing the burden of resistance across the continent.

## Graphical abstract

The incorporation of antibiotics into animal feeds is one of the drivers of antibiotic resistance, a global One Health challenge. To reduce the burden of antibiotic resistance, the use of alternative growth promoters and drugs must be promoted, as well as sensitization and implementation of regulations.

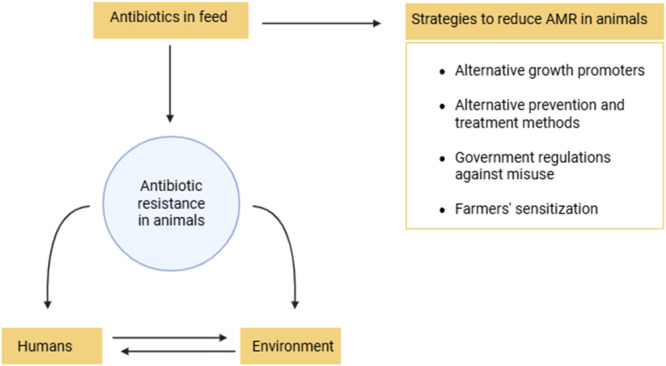

## 1 Introduction

The growth of animals is measured as an increase in the value of their body weight. Animal growth is usually rapid in their early life, declines gradually until puberty and reduces more as they grow into maturity (Hutu et al. 2020). Therefore, proper feeding is necessary at the different stages of growth, and feed formulation is often influenced by the weight of the animal, its weight at maturity, its composition gain and the average daily gain (i.e., growth rate) (Madikadike et al. 2022). All nutrients in the feed interact to influence animal growth, development, and health; hence, a proper diet is essential for animal growth as well as avoiding and treating infectious illnesses (Wu 2022).

Antibiotics are used in the treatment of bacterial infections in animals and to prevent the occurrence of diseases (Odey et al. 2023). They are usually included in animal feed formulations in subtherapeutic doses as growth‐promoting agents to boost animal weight and product yield, especially in meat production (Odey et al. 2023). Globally, commercial food products from animals fed with antibiotics‐supplemented meals sell at about 8% lower than products from animals fed with antibiotics‐free feed (Markovic et al. 2019). This relatively lower selling price increases the demand for such products and encourages animal producers to use antibiotics to stimulate the growth and yield of animals, thus establishing the use of antibiotics in animal production. Greater feed utilization efficiency, increased appetite, and enhanced skin and hair quality are advantages of antibiotics on farm animals (Rahman et al. 2022).

The extensive use of antibiotics in the production and health of animals has been associated with antibiotic resistance (Fomnya et al. 2021; Pandey et al. 2024; Drugea et al. 2025; Farrukh et al. 2025). This allows resistant bacteria in animals to spread to humans through contaminated food, water, and manure used as fertilizer (Ma et al. 2021). It has been reported that foods from animal sources contain a large number of resistant bacteria (Lerminiaux and Cameron 2019). In Africa, Western sub‐Saharan Africa has been reported to have the greatest burden of antibiotic resistance, and this subtly highlights it as a potential pandemic (Godman et al. 2022). Beyond Africa, the World Health Organization (WHO) reported a rising global trend in antibiotic resistance in 2022. To combat this growing threat, it is crucial to address the factors driving antibiotic resistance both in African countries and worldwide. Antibiotic resistance has been identified as one of the most significant emerging environmental issues, making it a global One Health challenge. The One Health approach is an important concept that recognizes the inseparable links between human, animal, and environmental health (Wang et al. 2021). Though research on antibiotic resistance has mostly concentrated on humans and animals, environmental factors play a crucial role in controlling antibiotic resistance. Antibiotic residues and resistance genes remain in the environment because of the frequent occurrence of antibiotic residues in water sources and other environmental areas (Yuan et al. 2023).

Some countries have implemented laws restricting the use of antibiotics in feed formulations to curb overuse and mitigate the emergence of antibiotic resistance (Ghimpețeanu et al. 2022). Nonetheless, antibiotics are still widely used in animals for growth enhancement, prophylactic, and therapeutic purposes in many countries, leading to concerns about antibiotic residues in animal products (Treiber and Beranek‐Knauer 2021). Residues can also contaminate groundwater and soil, posing significant risks to both human and animal health (Mehdi et al. 2018). This underscores the need for alternative solutions, as maintaining optimal animal product yield remains essential. Natural feed additives, such as probiotics and phytobiotics, present promising alternatives for promoting animal growth while mitigating the risks associated with antibiotic use (Fonseca et al. 2024). This review explores the impact of antibiotic use in animal feed on the rising rates of antibiotic resistance in Africa. It highlights antibiotic resistance as a critical One Health concern and proposes alternative growth promoters for sustainable animal production. Also, it discusses the potential for regulatory measures to curb antibiotic misuse in animal farming across the continent, aiming to mitigate the spread of antibiotic resistance.

## 2 Methodology

The articles referenced in this review were sourced from reputable scientific databases, including Google Scholar, Web of Science, ScienceDirect, and ResearchGate, to ensure the collection of high‐quality data. A total of 201 articles were included after excluding those that did not directly address the use of antibiotics in animal feed and the resulting antibiotic resistance in bacteria affecting both animals and humans. Articles reviewed included those addressing the use and misuse of antibiotics in livestock farming in Africa, reports of antibiotic resistance in bacteria in animals and humans in Africa, the route of transfer of antibiotic resistance and national legislations against the misuse of antibiotics in livestock farming. Keywords included “antibiotics in animal feed,” “bacterial resistance in livestock,” “bacterial resistance in humans,” “mechanisms of antibiotic resistance transfer,” “government regulations against antibiotic misuse in Africa,” “alternative growth promoters,” and “phytochemicals as animal feed additives.”

## 3 Antibiotic Use in Livestock of Africa

Antibiotics have been widely used in livestock for disease control and prevention as well as treatment of infections in animals (Liu et al. 2021; Kupczyński et al. 2024; Oh et al. 2025). Medicated feed refers to a homogeneous mixture of animal feed and veterinary medicinal products, serving as an oral route for administering veterinary drugs. Studies indicate that many animal feeds are supplemented with antibiotics from various classes (Table 1) to prevent diseases. Nevertheless, inconsistencies in usage, dosage, and frequency of administration raise significant health concerns (Ghimpețeanu et al. 2022).

**Table 1:** Classes of antibiotics used in livestock in Africa (Hosain et al. 2021; Mann et al. 2021; Odey et al. 2023).

Class	Examples
Aminoglycosides	Amikacin, gentamicin, kanamycin, neomycin, streptomycin
β‐lactams	Amoxicillin, benzylpenicillin, ceftiofur, cloxacillin, penicillin
Fluoroquinolones	Ciprofloxacin, danofloxacin, enrofloxacin, norfloxacin oxolinic acid
Glycolipids	Bambermycin
Ionophores	Monensin, narasin
Macrolides and lincosamides	Azithromycin, erythromycin, lincomycin, tilmicosin, tulathromycin, tylosin
Phenicols	Chloramphenicol, florfenicol
Polymyxins	Colistin
Polypeptides	Bacitracin
Streptogramins	Virginiamycin
Sulfonamides	Sulfamethoxazole
Tetracyclines	Chlortetracycline, doxycycline, tetracycline, oxytetracycline

Although reports on antibiotic use in animal production across Africa remain limited, the available data provide valuable insight into its widespread application. Between 2015 and 2019, data from 13 to 27 African countries indicated that antimicrobial use in animals ranged from 3,558 to 4,279 tonnes, with tetracyclines and polypeptides accounting for the largest proportion. Cattle and poultry production were reported to have the highest consumption of antibiotics in Africa (Mshana et al. 2021). Reports on the sales of antibiotics for animal use stated an estimated mean quantity of 1,538,443 kg sold in South Africa and 23,234 kg in South‐western Nigeria over a period of 3 years, while Zambia reported a mean quantity of 41,280.87 kg in 1 year (Azabo et al. 2022). The study also highlighted that tetracyclines, beta‐lactams, aminoglycosides, and fluoroquinolones are the commonly reported antibiotics in these regions.

In Nigeria, antibiotic use in animal production is significant. A study conducted on antibiotic use among poultry farmers in Oyo State (South‐west) and Plateau State (North‐central) in Nigeria revealed that 98% of poultry farmers administered antibiotics as a prophylactic treatment to day‐old chicks (Ndahi et al. 2023). The study also reported the use of 351 kg of active ingredients from seven different classes of antibiotics, including tetracyclines, penicillins, aminoglycosides, polypeptides, fluoroquinolones, amphenicols, and macrolides. Although there has been some progress in reducing antibiotic use in animal production in South Africa, tetracyclines, aminoglycosides, and penicillins are still widely used (Kimera et al. 2020). Similar patterns have been observed in other African countries, including Tanzania, Cameroon, Zambia, Ghana, and Egypt (Kimera et al. 2020). Across the different countries, tetracyclines, aminoglycosides, and penicillin use has been extensively reported and can be attributed to the availability of these classes of antibiotics even in rural communities and uneducated farmers.

Aquaculture contributes largely, approximately 15%, to the protein needs of people worldwide (Larcombe et al. 2025). In Africa, fish farmers use antibiotics for the prevention and treatment of disease in fishes and also as feed additives for growth promotion (Limbu 2020; Kilusungu et al. 2024). These antibiotics are not all metabolized and are excreted with urine and faeces (Moffo et al. 2024), and some classes such as quinolones bioaccumulate in fish tissues, which is detrimental to human health, as it contributes to the development of antibiotic resistance in both fish and humans (Yang et al. 2020).

When antibiotics are used responsibly for the treatment of sick animals, they reduce the pressure for the selection of resistance (Odey et al. 2023). Studies in Nigeria indicate the common use of antibiotics in treating sick animals. For instance, tetracycline, tylosin, and gentamicin are used to treat sick pigs in Ogun state (Adebowale et al. 2020), while tetracycline, chloramphenicol, and metronidazole are used for sick birds in the same state (Adebowale et al. 2022). In Ghana, tetracycline, neomycin, tylosin, streptomycin, and colistin are used to treat sick birds (Paintsil et al. 2021). In Ethiopia, tetracycline, aminoglycosides, and sulfonamide‐trimethoprim are used for treating cattle, sheep, goats, equines, and poultry (Gemeda et al. 2020). In Tanzania, tetracycline, penicillin, sulfonamides, macrolides, and quinolones are commonly used to treat sick poultry, pigs, and ruminants (Mdegela et al. 2021). Kenya reports the use of tylosin, erythromycin, colistin, and neomycin for treating sick poultry birds (Kiambi et al. 2021). A study in Malawi highlights the frequent use of tetracycline, streptomycin, and erythromycin to treat poultry, pigs, and goats (Mankhomwa et al. 2022).

Antibiotics are also used in disease prevention for animals that are perceived to be at risk even in the absence of clinical symptoms (Odey et al. 2023). In the Ashanti region of Ghana, 97% of commercial poultry farmers and 43% of domestic poultry farmers used antibiotics prophylactically in animal production (Paintsil et al. 2021). About 84% of farmers in Southern Togo consented to the use of antibiotics for prophylaxis in the production of poultry and pigs (Bedekelabou et al. 2022). A study in Ethiopia found that 10% of farmers used antibiotics for prophylaxis (Gemeda et al. 2020), while another reported that 39.1% of farmers administered antibiotics for both prophylaxis and metaphylaxis in poultry, cattle, sheep, and goats (Gebeyehu et al. 2021). In Tanzania, 60% of farmers reportedly use antibiotics to prevent diseases in poultry and pig farming (Mdegela et al. 2021).

Antibiotics are also used as feed additives in subtherapeutic doses to promote growth (Rahman et al. 2022). The exact mechanism behind this effect is not fully understood, but it is suggested that antibiotics promote growth by influencing gut microbiota and altering physiological processes in animals (Low et al. 2021). The growth‐promoting effect of antibiotics may be due to a reduction in energy expended by the animals on maintaining their gastrointestinal commensal bacteria, leading to an increase in the overall energy available for animal growth. Antibiotics also function as growth promoters by reducing growth‐suppressing metabolites such as bile degradation products (Islam et al. 2022). An example is the alteration in the level of cholyltaurine hydrolase (bile acid‐transforming enzymes) activity in the gut, which leads to increased weight gain in the animals (Low et al. 2021).

Reports indicate the use of various antibiotics as growth promoters across different countries. In Tanzania, tetracycline, tylosin, and streptomycin have been used in poultry, cattle, and goats (Caudell et al. 2020), while oxytetracycline, cloxacillin, sulfamethoxazole, tylosin, and norfloxacin have been reported in poultry, pigs, cattle, sheep, and goats (Mdegela et al. 2021). In Kenya, tetracycline, tylosin, erythromycin, neomycin, and colistin have been used in poultry (Kiambi et al. 2021). Similarly, in Cameroon, tetracycline, amoxicillin, colistin, and enrofloxacin have been employed in poultry (Moffo et al. 2024). In Malawi, tetracycline, streptomycin, erythromycin, and colistin have been used as growth promoters in poultry, goats, and pigs. Antibiotics have also been used as growth promoters in pigs in South‐western Nigeria (Adebowale et al. 2020) as well as by poultry farmers in Ghana (Paintsil et al. 2021). Studies have shown that chickens feeds containing tetracycline fermentation by‐products exhibited growth‐promoting effects (Gonzalez Ronquillo and Angeles Hernandez 2017).

The projected population increase in Africa to 2.4 billion by 2025 is expected to place greater demand on the agriculture sector. As a result, the use of antibiotics at subtherapeutic doses in animal production will likely continue to be favored to meet the growing demand for meat from the rising population (Hosain et al. 2021). The continuous use of antibiotics, even at subtherapeutic doses, in animals creates selective pressure that facilitates the propagation of antibiotic‐resistant commensal and pathogenic bacteria through natural selection (Bava et al. 2024). These antibiotic‐resistant bacteria evade the effects of antibiotics by mechanisms such as deactivation of antibiotics, use of efflux pumps, and modification of target sites (Belay et al. 2024). The resistance genes can then be transferred between bacteria either vertically or horizontally (Patangia et al. 2022), raising significant concerns for both animal and human health. Nevertheless, the need for quality animal production remains crucial, underscoring the importance of identifying alternative methods to enhance livestock productivity.

## 4 Antibiotic Resistance in Bacteria From Farmed Animals

Antibiotic resistance in bacteria from animals is widespread across Africa, with a diverse range of resistance genes identified against multiple major antibiotic classes (Table 2). Animals are hosts to a diversity of antibiotic‐resistant bacteria and genes (Xu et al. 2022), with resistance observed against major antibiotic classes including aminoglycosides (aminoglycoside acetyltransferase, *aac;* aminoglycoside phosphotransferase, *aph;* aminoglycoside adenylyltransferase, *ant*), β‐lactams (β‐lactamase genes, *bla;* common β‐lactamase types, *bla*_*TEM*_, *bla*_*SHV*_, *bla*_*CTX‐M*_), colistin (mobilized colistin resistance, *mcr*), amphenicol, chloramphenicol (chloramphenicol acetyltransferase, *cat*), florfenicol (*floR*), fluoroquinolone and quinolone (fluoroquinolone acetyltransferase, *fca;* mutations in the gyrase genes, *gyrA, gyrB;* mutations in the topoisomerase IV genes, *parC, parE;* plasmid‐mediated quinolone resistanc*e, qnr*), macrolide‐lincosamide‐streptogramin B (erythromycin ribosome methylase, *erm*), sulfonamides (dihydropteroate synthetase, *sul*), tetracyclines (*tet*), vancomycin (*van*), and multidrug (various transporters or efflux pumps, *mdr*), respectively (He et al. 2020; Larsson and Flach 2022; Zhang et al. 2023; Akhlaghi et al. 2024).

**Table 2:** Antibiotic resistance reported in livestock in African countries (2018–2024).

Country	Animals	Resistant Organisms	Antibiotics tested	Resistance genes	References
Burkina Faso	Poultry	*Escherichia coli* Typhimurium	Ampicillin, chloramphenicol, streptomycin, sulfonamide, trimethoprim	*aadA1, bla*_*TEM‐1B*_, *catA1, dfrA1, strA, strB, sul1, sul2*,	Kagambèga et al. (2018)
Egypt	Poultry	*Klebsiella oxytoca, Klebsiella pneumoniae*	Amoxiclav, cefadroxil, cefotaxime, chloramphenicol, doxycycline, oxytetracycline, penicillin‐G, trimethoprim	*bla*_*CTX‐M*_, *bla*_*SHV*_, *bla*_*TEM*_	Abd El‐Tawab et al. (2022)
	Cattle	*Klebsiella pneumoniae*	Amikacin, amoxicillin‐clavulanic acid, ampicillin, azithromycin, aztreonam, cefepime, chloramphenicol, ciprofloxacin, imipenem, nalidixic acid, sulfamethoxazole‐trimethoprim, tetracycline	*—*	Ammar et al. (2021)
Ethiopia	Poultry	*Klebsiella pneumoniae*	Cefixime, nalidixic acid, oxytetracycline, penicillin, sulphamethoxazole, tetracycline	*—*	Kahin et al. (2024)
	Cattle	*Escherichia coli*	Amikacin, amoxicillin‐clavulanic acid, ampicillin, ceftriaxone, cephalothin, chloramphenicol, ciprofloxacin, gentamicin, nalidixic acid, streptomycin, sulfamethoxazole‐trimethoprim, sulfisoxazole, tetracycline	*—*	Tadesse et al. (2024)
Gabon	Pig, poultry	Enterococci	Ampicillin, cephalothin, ciprofloxacin, erythromycin, kanamycin, rifampicin, streptomycin, streptomycin, teicoplanin, tetracycline, vancomycin	*—*	Desire et al. (2022)
Ghana	Cattle, goat, pig, poultry, sheep	*Enterococcus* species	Ampicillin, chloramphenicol, ciprofloxacin, erythromycin, linezolid, tetracycline, vancomycin	*aac(6′)‐aph(2″), aac(6′)‐Ii, aph(3′)‐III, ant(6)‐Ia, cat, dfrG, ermB, ermT, lnuB, lsaA, lsaA, lsaE, lsaE, msrC, msrC, tetL, tetM, tetS*	Amuasi et al. (2023)
Kenya	Goat	*Enterobacter* species, *Escherichia coli*, *Escherichia vulneris, Klebsiella oxytoca*,	Amoxicillin‐clavulanic acid, azithromycin, cefotaxime, cefuroxime, ciprofloxacin, meropenem, nitrofurantoin, oxacillin	*bla*_*TEM*_	Kabui et al. (2024)
	Goat	Coagulase negative Staphylococci, *Staphylococcus aureus*,	Azithromycin, ciprofloxacin, nitrofurantoin, oxacillin	*mecA*	Kabui et al. (2024)
	Fish	*Proteus species, Pseudomonas aeruginosa, Staphylococcus aureus, Vibrio cholerae, Vibrio parahaemolyticus*	Ampicillin/cloxacillin, cefpodoxime, ceftazidime, meropenem, penicillin G, rifampicin, streptomycin, vancomycin	*aadA, bla*_*CMY‐2*_, *bla*_*TEM‐1*_, *dfrA7, strA, sulII, tetA, tetC*	Mumbo et al. (2023)
Malawi	Goat	*Bacillus* species, *Citrobacter* species, *Escherichia coli*, *Staphylococcus aureus*, *Streptococcus* species	Ampicillin, erythromycin, penicillin, streptomycin, tetracycline	*—*	Kalumbi et al. (2022)
	Cattle	*Bacillus* species, *Citrobacter* species, *Klebsiella* species, *Staphylococcus aureus*	Ampicillin, chloramphenicol, gentamicin, tetracycline	*—*	Kalumbi et al. (2022)
Nigeria	Poultry	*Enterococcus* species	Ampicillin, chloramphenicol, ciprofloxacin, erythromycin, fosfomycin, nitrofurantoin, penicillin G, rifampin, vancomycin	*—*	Isichei‐Ukah et al. (2024)
	Pig	*Escherichia* coli, *Klebsiella pneumoniae, Proteus mirabilis, Yersinia enterocolitica*	Amoxicillin, ampicillin, ceftazidime, ceftriaxone, ertapenem, gentamicin, norfloxacin, pefloxacin, tetracycline	*—*	Ajayi et al. (2023)
	Fish	*Aeromonas hydrophila, Bacillus subtilis, Citrobacter freundii, Corynebacterium aquaticum, Escherichia coli, Klebsiella pneumoniae, Proteus mirabilis, Pseudomonas aeruginosa, Salmonella enterica, Shigella* species, *Staphylococcus aureus, Streptococcus agalactiae*	Ampicillin, ciprofloxacin, florfenicol, gentamicin, oxacillin, oxytetracycline, penicillin, streptomycin, tetracycline, vancomycin	*—*	Adah et al. (2022)
	Poultry	*Escherichia coli, Salmonella* species	Amoxicillin‐clavulanic acid, cephalexin, ciprofloxacin, gentamicin, nalidixic acid, ofloxacin, pefloxacin, penicillin, streptomycin, sulfamethoxazole‐trimethoprim	*—*	Bamidele et al. (2022)
	Poultry	*Salmonella* species	Flumequine, pefloxacin, penicillin	*—*	Oloso et al. (2019)
	Poultry	*Escherichia coli*	Dihydrofolate, phenicols, quinolones, sulphonamides, tetracycline	*aac, bla*_*CMY‐2*_, *bla*_*CTX‐M*_, *bla*_*SHV*_, *bla*_*TEM*_, *bla*_*VEB*_, *tetA*	Chah et al. (2018)
Rwanda	Cattle, goat, pig, poultry	*Escherichia coli*	Amoxicillin‐clavulanic acid, ampicillin, azithromycin, cefoxitin, ceftriaxone, chloramphenicol, ciprofloxacin, colistin, nalidixic acid, streptomycin, tetracycline	*—*	Manishimwe et al. (2021)
Senegal	Poultry	*Salmonella* species	Ciprofloxacin, erythromycin, nalidixic acid, ofloxacin, sulfamethoxazole‐trimethoprim	*aac, aadA7, aph, bla*_*TEM*_, *dfrA1, dfrA14, dfrA15, fosA, gyrA, parC, qnr, sul1, sul2, tetA, tetB*	Dieye et al. (2022)
South Africa	Pig	*Mammaliicoccus sciuri, Staphylococcus aureus, Staphylococcus epidermidis, Staphylococcus hyicus*	Ceftaroline, clindamycin, erythromycin, fusidic acid, levofloxacin, oxacillin, sulfamethoxazole‐trimethoprim, tetracycline	*ermC, inuA, inuG, mecA, mecC, tet45, tetK, tetL, tetM, tetT*	Ocloo et al. (2024)
	Pig	*Escherichia coli*	Amikacin, amoxicillin‐clavulanic acid, ampicillin, cefepime, cefotaxime, cefoxitin, ceftazidime, ceftriaxone, cephalexin, chloramphenicol, ciprofloxacin, gentamicin, imipenem, meropenem, nalidixic acid, piperacillin‐tazobactam, sulfamethoxazole‐trimethoprim, tetracycline, tigecycline	*—*	Abdalla et al. (2021)
	Poultry	*Salmonella* species	Amoxicillin‐clavulanic acid, ampicillin, cefotaxime, ceftazidime, chloramphenicol, ciprofloxacin, doxycycline, erythromycin, gentamicin, kanamycin, nalidixic acid, norfloxacin, oxytetracycline, spectinomycin, streptomycin, sulfamethoxazole‐trimethoprim	*—*	Mokgophi et al. (2021)
	Cattle, goats, horses, pigs, poultry, sheep	*Salmonella* species	Ampicillin, chloramphenicol, ciprofloxacin, erythromycin, gentamicin, kanamycin, streptomycin, tetracycline	*ampC, bla*_*CTX‐M*_, *bla*_*OXA*_, *bla*_*SHV*_, *bla*_*TEM*_, *catI, catII, mcr‐1, mecA, qnrA, qnrD*	Ramatla et al. (2021)
	Pig	*Staphylococcus aureus*	amikacin, Ampicillin, cefoxitin, chloramphenicol, ciprofloxacin, clindamycin, doxycycline, erythromycin, gentamicin, levofloxacin, linezolid, moxifloxacin, nitrofurantoin, penicillin‐G, rifampicin, sulfamethoxazole‐trimethoprim, teicoplanin, tetracycline, tigecycline, vancomycin	*aac(6′)‐aph(2″), blaZ, ermC, mecA, msrA, tetK, tetM*	Sineke et al. (2021)
	Pig	*Campylobacter* species	Ampicillin, ciprofloxacin, erythromycin, gentamicin, nalidixic acid, streptomycin, tetracycline	*bla*_*OXA‐61*,_ *cmeB, tetO*	Sithole et al. (2021)
	Cattle	*Enterococcus* species	Amoxicillin, ampicillin, erythromycin, linezolid, penicillin, tetracycline, vancomycin	*vanA, vanB, vanC, tetK, tetL, msrA/B, mefA*	Tatsing Foka and Ateba (2019)
	Cattle	*Salmonella* species	Ampicillin, cefotaxime, cephalexin, enrofloxacin, erythromycin, kanamycin, oxytetracycline, rifampicin	*invA, spvC*	Dlamini et al. (2018)
South Sudan	Poultry	*Salmonella* species	Ampicillin, tetracycline	*—*	Saad et al. (2022)
Tanzania	Cattle	*Escherichia coli*	Ampicillin, cefotaxime, chloramphenicol, ciprofloxacin, gentamicin, nalidixic acid, sulfamethoxazole‐trimethoprim, tetracycline	*—*	Azabo et al. (2022)
	Cattle, pigs, poultry	*Escherichia coli*	Aminoglycoside, cephalosporin, clavam, dihydrofolate, glycopeptides, penicillin, quinolones, sulphonamide, tetracycline	*bla*_*CTX‐M*_, *sulIII, tetW*	Katakweba et al. (2018)
	Fish	*Campylobacter* species, *Escherichia coli, Listeria* species, *Salmonella* species, *Shigella* species, *Staphylococcus* species	Azithromycin, ciprofloxacin, erythromycin, gentamicin, penicillin	*—*	Marijani (2022)
Togo	Pigs	*Escherichia coli*	Amikacin, amoxicillin‐clavulanic acid, ampicillin, aztreonam, cefalothin, cefepime, cefotaxime, cefoxitin, ceftazidime, chloramphenicol, ciprofloxacin, gentamicin, nalidixic acid, ofloxacin, piperacillin, ticarcillin, trimethoprim	*—*	Atrah et al. (2024)
	Cattle, pigs, poultry	*Escherichia coli*	Ampicillin, cefotaxime, ceftazidime, chloramphenicol, ciprofloxacin, erythromycin, gentamicin, tetracycline, trimethoprim	*—*	Munengwa et al. (2022)
Tunisia	Poultry	*Campylobacter* species	Amoxicillin‐clavulanic acid, ampicillin, chloramphenicol, ciprofloxacin, erythromycin, gentamicin, nalidixic acid, tetracycline	*aphA‐3, bla*_*OXA‐61*_, *cmeB, ermB, gyrA, tetA, tetB, tetL, tetO*	Béjaoui et al. (2022)
	Poultry	*Campylobacter* species	Macrolides, phenicols, quinolones, tetracyclines	*—*	Gharbi et al. (2018)
Uganda	Poultry	*Escherichia coli*	Ampicillin, gentamicin, kanamycin, neomycin, sulphonamides, tetracycline, trimethoprim	*—*	Samuel et al. (2023)
	Cattle	*Escherichia coli*	Amoxicillin‐clavulanate acid, ampicillin, cefazolin, cefepime, ceftazidime, ceftriaxone, cefuroxime, ciprofloxacin, cotrimoxazole, ertapenem, imipenem, levofloxacin, nitrofurantoin, tetracycline	*—*	Iramiot et al. (2020)
	Cattle	*Klebsiella pneumoniae*	Amoxicillin‐clavulanic acid, cefazolin, cefepime, ceftazidime, ceftriaxone, cefuroxime, ciprofloxacin, cotrimoxazole, ertapenem, imipenem, levofloxacin, nitrofurantoin, tetracycline	*—*	Iramiot et al. (2020)
	Poultry	*Salmonella* species	Beta‐lactams, chloramphenicol, fluoroquinolones, sulphonamide, tetracycline, trimethoprim	*blaTEM, cmlA, dhfrI, dhfrVII, qnrS, sul1, tetA*	Odoch et al. (2018)
	Fish	*Aeromonas* species, *Plesiomonas shigelloides*	Ampicillin, ceftaxime, erythromycin, oxacillin, penicillin, streptomycin, tetracycline	*—*	Wamala et al. (2018)
Zimbabwe	Fish	*Bacillus cereus, Edwardsiella tarda, Escherichia coli, Listeria* species, *Proteus mirabilis, Staphylococcus aureus*	Cloxacillin, erythromycin, lincomycin, neomycin, rifampicin, streptomycin, tetracycline	*—*	Gufe et al. (2022)

Poultry farming presents a high risk for the development of antibiotic resistance, particularly in unregulated small‐scale enterprises, as observed in low‐income countries. Poultry farming is highly profitable and well‐suited to areas with limited space, which often leads to the extensive use of antibiotics to prevent infections. Resistance is more likely to develop in such environments due to overcrowding and poor sanitation (Ahmad et al. 2023). Resistant bacteria have also been recovered from livestock such as cattle, sheep, goats, camels, and pigs (Mshana et al. 2021; Hamame et al. 2022). Antibiotic residues have been found in animal manure, i.e., including fleroxacin in chicken, norfloxacin in chicken, ciprofloxacin in swine, chicken and cattle, enrofloxacin in swine, cattle and chicken, oxytetracycline in swine and cattle, chlortetracycline in swine and cattle, tetracycline in swine, sulphonamides in swine, chicken and cattle, macrolides in swine, and nitrofurans in swine, chicken and cattle (Yu et al. 2019; Ma et al. 2021).

## 5 Transfer of Antibiotic‐Resistant Bacteria From Animals to Humans

Antibiotic resistance is a global crisis with severe implications for human health. Infections caused by antibiotic‐resistant bacteria are difficult to treat, leading to increased mortality rates (Hetta et al. 2023). The WHO reports that approximately 80% of multidrug‐resistant bacterial infections result from antibiotic misuse (Khalil et al. 2021). By 2050, antibiotic resistance is projected to cause 10 million deaths annually, highlighting the urgent need for research interventions (O'Rourke et al. 2020). Patients with antibiotic‐resistant infections often do not respond to first‐line of antibiotics (Baciu et al. 2024), necessitating multiple or more potent drugs, which are often expensive, more toxic, and increase overall antibiotic exposure (Siedentop et al. 2024). Prolonged recovery times lead to extended treatments, escalating healthcare costs, burdening families, and reducing workforce productivity, thereby impacting the economy (Abban et al. 2023).

Literature reports highlight the alarming incidence of antimicrobial resistance in humans in Africa (Table 3). Bacteria, including *Acinetobacter baumannii, Escherichia coli, Enterococcus* species, *Klebsiella pneumoniae, Proteus* species, *Pseudomonas* species, *Salmonella* species, *Shigella* species, *and Staphylococcus aureus*, have been reported to resist multiple antibiotics such as amoxicillin, amoxicillin‐clavulanic acid, chloramphenicol, ciprofloxacin, gentamicin, ofloxacin, pefloxacin, sparfloxacin, and streptomycin (Kaapu et al. 2022; Ahmed et al. 2024; Sakalauskienė et al. 2025). Antibiotic resistance arises from multiple factors, primarily antibiotic resistance genes, inappropriate use of antibiotics, and extensive agricultural application (Nadgir and Biswas 2023). Mobile genetic elements such as plasmids, integrons, transposons, and integrative and conjugative elements facilitate horizontal transfer among microbes, especially pathogenic bacteria (Zhu et al. 2021).

**Table 3:** Antimicrobial resistance in bacteria obtained from human clinical samples in African countries (2019–2023).

Country	Sample	Resistant bacteria	Antibiotics tested	Resistance genes	References
Congo	Urine	*Escherichia coli*, *Staphylococcus aureus, Streptococcus* species	Amoxicillin/clavulanic acid, ceftriaxone, ciprofloxacin, doxycycline, gentamicin, meropenem, nalidixic acid	*—*	Bunduki et al. (2023)
	Stool	*Escherichia coli*	Amoxicillin, amoxicillin–clavulanic acid, azithromycin, ceftazidime, ciprofloxacin, nalidixic acid, ofloxacin, piperacillin–tazobactam	*—*	Mfoutou Mapanguy et al. (2021)
	Gastric biopsy specimens	*Helicobacter pylori*	Amoxicillin, clarithromycin, Levofloxacin, metronidazole	*—*	Tshibangu‐Kabamba et al. (2020)
Egypt	Blood cultures, urine, wound swabs	*Acinetobacter* species, *Enterobacter* species, *Klebsiella* species, *Proteus* species, *Pseudomonas* species	Amikacin, amoxicillin/clavulanate, ampicillin/sulbactam, cefepime, cefepime, cefoxitin, cefpodoxime, ceftazidime, ceftriaxone, ciprofloxacin, colistin, doxycycline, gentamicin, imipenem, levofloxacin, meropenem, nitrofurantoin, piperacillin/tazobactam, tetracycline, tobramycin, trimethoprim‐sulfamethoxazole	*—*	Fahim (2021)
	—	*Pseudomonas aeruginosa*	Amoxicillin–clavulanic acid, ampicillin/sulbactam, azlocillin, carbenicillin, cefepime, ciprofloxacin, colistin sulfate, levofloxacin, polymyxin B, tigecycline	*bla*_*CTX‐M‐15*_, *bla*_*GIM*_, *bla*_*IMP*_, *bla*_*SPM*_, *bla*_*VIM*_	Farhan et al. (2019)
Ethiopia	—	*Acinetobacter* species, coagulase negative Staphylococci, *Enterococci* species, *Escherichia coli, Klebsiella pneumoniae, Proteus mirabilis, Pseudomonas aeruginosa, Serratia* species, *Staphylococcus aureus, Streptococcus pyogenes*	Amikacin, amoxicillin‐clavulanic acid, ampicillin, cefotaxime, ceftazidime, ceftriaxone, chloramphenicol, ciprofloxacin, cotrimoxazole, erythromycin, gentamicin, meropenem, nitrofurantoin	*—*	Hailemariam et al. (2021)
	Urinary tract and surgical site	*Acinetobacter baumannii*, *Pseudomonas aeruginosa*	Ampicillin, ciprofloxacin, meropenem, piperacillin	*—*	Motbainor et al. (2020)
Mozambique	Urine and blood	*Escherichia coli*	Ciprofloxacin, gentamicin, trimethoprim‐sulfamethoxazole	*bla*_*CTX‐M*_, *bla*_*SHV*_, *bla*_*TEM*_	Estaleva et al. (2021)
Nigeria	Urine	*Pseudomonas aeruginosa*	Amoxicillin, amoxicillin‐clavulanic acid, chloramphenicol, ciprofloxacin, gentamicin, ofloxacin, pefloxacin, septrin, sparfloxacin, streptomycin	*—*	Alabi et al. (2021))
	Urine	*Enterococcus, Klebsiella, Proteus, Pseudomonas, Staphylococcus*	Amoxicillin, amoxicillin‐clavulanic acid, ampicillin, ciprofloxacin, erythromycin, nitrofurantoin	*—*	Nwana et al. (2020)
	Stool	*Escherichia coli*	amoxicillin, Ampicillin, cefotaxime, cefoxitin, ceftazidime, ciprofloxacin, gentamicin, imipenem, streptomycin, trimethoprim	*bla*_*CTX‐M*_, *bla*_*SHV*_, *bla*_*TEM*_, *ampC*	Zakou et al. (2020)
South Africa	Wounds	*Acinetobacter baumannii, Escherichia coli, Klebsiella pneumoniae, Pseudomonas aeruginosa*	Amoxicillin, ampicillin, cefepime, ciprofloxacin, gentamicin, tigecycline, trimethoprim sulfamethoxazole	*—*	Kaapu et al. (2022)
	Stool	*Escherichia coli*	Ampicillin, doxycycline, erythromycin, penicillin G, tetracycline, trimethoprim	*ampC, bla*_*TEM*,_ *sulII, tetA*	Mkuhlu et al. (2020)
	Urine	*Neisseria gonorrhoeae*	Azithromycin, ciprofloxacin, penicillin, tetracycline	*bla*_*TEM*_, *gyrA, parC, tetM*	Maduna et al. (2020)
Zambia	Blood	*Citrobacter freundii, Enterobacter aerogenes, Enterobacter agglomerans, Enterobacter cloacae, Escherichia coli, Klebsiella oxytoca, Klebsiella pneumoniae, Proteus* mirabilis, *Serratia marcescens, Staphylococcus aureus*	Ampicillin, chloramphenicol, cotrimoxazole, nalidixic acid, penicillin, tetracycline	*—*	Mwansa et al. (2022)

**Figure 1:**
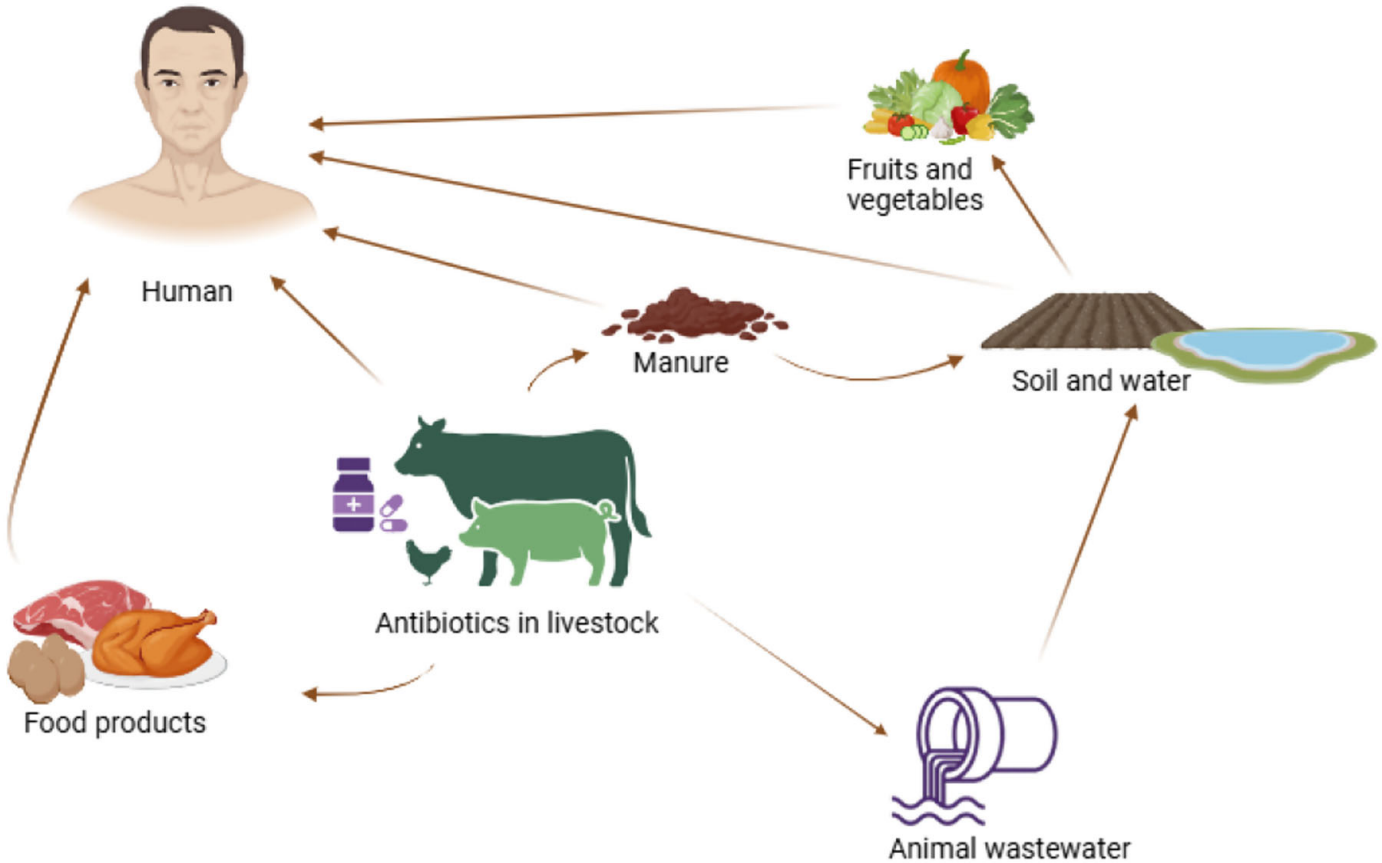
Antibiotic resistance transmission routes from bacteria in food animals and farm/food production environments to human‐associated bacteria (created with Biorender). Aligned with the One Health framework, this figure highlights the interconnected pathways through which antibiotic‐resistant bacteria and resistance genes can disseminate across environmental, animals and human compartments, emphasizing the heightened risk of transfer to humans due to these complex interactions.

The misuse of antibiotics in animal production contributes to antibiotic‐resistant bacteria in food animals (Figure 1), impacting animal health and potentially leading to resistant infections in humans (Ma et al. 2021). Prolonged antibiotic use in livestock promotes the emergence and spread of resistant strains, which can be transferred to humans through various routes (Figure 1), posing a significant challenge to clinical antimicrobial therapy (Ma et al. 2021).

Antibiotic‐resistant bacteria and genes can transfer from animals to humans through multiple routes (Table 4), including direct contact with animals, exposure to faeces or manure, inhalation of contaminated bioaerosols, wastewater exposure, and consumption of contaminated animal products (Mshana et al. 2021; Xu et al. 2022). Individuals in close contact with animals, such as farm workers, slaughterhouse employees, and veterinarians, are particularly vulnerable (Ma et al. 2021; Oludairo et al. 2023). Antibiotic‐resistant bacteria excreted in animals' faeces and urine contaminate the environment, facilitating further transmission (Zhao et al. 2023). The use of animal manure introduces resistant bacteria contained into soils, increasing exposure risks for farm workers (Marutescu et al. 2022). Farm effluent and treated wastewater serve as reservoirs for antibiotic‐resistant bacteria and resistance genes, posing a public health threat when applied to agricultural lands (Ibekwe et al. 2023).

**Table 4:** Isolation of antibiotic‐resistant bacteria from food products and environmental samples (2020*–*2024).

Country	Source	Bacterial isolates	Antibiotics tested	Resistance genes	Reference
Algeria	Eggs	*Salmonella* species	Amoxicillin, ampicillin, clavulanic acid, erythromycin, nalidixic acid	*—*	Merati and Boudra (2023)
Botswana	Effluent, beetroot and spinach	*Campylobacter* species, lactose fermenters, *Listeria* species, non‐lactose fermenters, *Pseudomonas* species	Ampicillin, erythromycin, penicillin, streptomycin, vancomycin	*aac, aad, ampC, aph, bla*_*CTX‐M*_, *bla*_*OXA‐663*_, *bla*_*SHV*_, *bla*_*TEM‐122*_, *bla*_*TEM‐1B*_, *cfxA6, drf, erm, mph, qnr, tet*	Brooks et al. (2023)
Cameroon	Abattoir workers, pigs	Extended spectrum beta lactamase *Escherichia coli*	Amoxicillin‐clavulanic acid, cefixime, cefotaxime, ceftazidime, cefuroxime, chloramphenicol, ciprofloxacin, fosfomycin, gentamicin, tetracycline, trimethoprim‐sulfamethoxazole	*bla*_*CTX‐M*_, *bla*_*TEM*_, *bla*_*SHV*_	Matakone et al. (2024)
	Cattle faeces	*Escherichia coli*, *Proteus* species, *Providencia* species	Imipenem, meropenem	*—*	Bissong et al. (2023)
	Raw milk	*Staphylococcus aureus*	Ampicillin, azithromycin, cefotaxime, ceftriaxone, chloramphenicol, ciprofloxacin, cotrimoxazole, gentamicin, nalidixic acid, nitrofurantoin, norfloxacin, oxacillin, tetracycline, vancomycin	*—*	Banu and Zewdu Geberemedhin (2022)
	Abattoir waste, abattoir wastewater, butchering table, slaughter slab	*Escherichia coli*, *Salmonella* species, *Shigella* species	Amoxicillin‐clavulanic acid, ampicillin, ceftriaxone, chloramphenicol, gentamicin, nalidixic acid, streptomycin, tetracycline	*—*	Esemu et al. (2022)
Ethiopia	Raw fish	*Escherichia coli*	Chloramphenicol, erythromycin, streptomycin, tetracycline, trimethoprim‐sulfamethoxazole	*—*	Yohans et al. (2022)
	Raw cattle at butcher house	Extended spectrum beta lactamase *Salmonella* species	Amoxicillin‐clavulanic acid, cefotaxime, ceftazidime	*—*	Worku et al. (2022)
	Camel, goats, raw milk of cows	*Escherichia coli*	Ceftriaxone, doxycycline, penicillin, spectinomycin, vancomycin	*—*	Balemi et al. (2021)
Ghana	Fish farms	*Athrobacter* species, *Cellobiococcus* species, *Citrobacter diversus, Citrobacter freundii, Edwardsiella tarda, Enterobacter aerogenes, Escherichia coli, Klebsiella oxytoca, Klebsiella pneumoniae, Micrococcus* species, *Proteus mirabilis, Salmonella enterica, Salmonella paratyphi ‘A’, Salmonella* species, *Serratia marcescens, Shigella sonnie, Staphylococcus* species, *Streptococcus* species	Ampicillin, cefotaxime, ceftriaxone, cefuroxime, chloramphenicol, cotrimoxazole, gentamicin, tetracycline	*bla*_*CTX‐M*_, *bla*_*EBC*_, *bla*_*mecA*_, *bla*_*TEM*_, *bla*_*TEM‐1*_, *catI, cmlA, gyrA, qnrB, qnrS, sulI, sulIII, tetB*	Agbeko et al. (2022)
	Raw meat at slaughter sites	Extended spectrum beta lactamase *Escherichia coli*	Amikacin, ampicillin, cefotaxime, ceftriaxone, cefuroxime, chloramphenicol, ciprofloxacin, gentamicin, meropenem, sulfamethoxazole‐trimethoprim, tetracycline	*bla*_*TEM*_	Dsani et al. (2020)
Kenya	Abattoir workers	*Staphylococcus aureus*	Cefoxitin, ciprofloxacin, clindamycin, erythromycin, gentamicin, linezolid, oxacillin, penicillin, sulfamethoxazole‐trimethoprim, tetracycline, trimethoprim	*aacA‐aphD, blaZ, dfrA, dfrG, ermC, mecA, tetK, tetM*	Obanda et al. (2022)
Libya	Dairy product and milk	*Salmonella enterica*	Amoxicillin, amoxicillin‐clavulanic acid, ampicillin, bacitracin, chloramphenicol, clindamycin, cloxacillin, erythromycin, lincomycin, methicillin, nitrofurantoin, oxytetracycline, penicillin G, sulfamethoxazole‐trimethoprim, tetracycline, vancomycin	*—*	Garbaj et al. (2022)
Morocco	Table eggs	*Escherichia coli, Salmonella* species	Ciprofloxacin, kanamycin, nalidixic acid, tetracycline	*—*	El Ftouhy et al. (2023)
	Broiler chicken meat	*Staphylococcus aureus*	Erythromycin, tetracycline, trimethoprim‐sulfamethoxazole	*—*	Nacer et al. (2022)
	Broiler chicken meat	*Salmonella* species	Ciprofloxacin, kanamycin, nalidixic acid, tetracycline, trimethoprim‐sulfamethoxazole	*—*	Nacer et al. (2022)
Nigeria	Chicken	Methicillin‐resistant *Staphylococcus aureus*	Amikacin, azithromycin, ceftaroline, chloramphenicol, ciprofloxacin, clarithromycin, clindamycin, doxycycline, erythromycin, gentamicin, kanamycin, levofloxacin, linezolid, minocycline, moxifloxacin, nitrofurantoin, penicillin G, rifampin, sulfonamides, tetracycline, trimethoprim, trimethoprim, trimethoprim‐sulfamethoxazole	*coa, eta, etb, hla, hlb, icaA, icaB, pvl, sea, seb, sec, sed, sel, sep, sev, spa, tsst*	Igbinosa et al. (2023)
	Fermented milk product	*Ent. Thailandicus, Lact. Delbrueckii, Lent. senioris, Leuc. Pseudomesenteriodes, Limosilactobacillus ermentum, Streptococcus infantarius, Streptococcus thermophilus*	Ciprofloxacin, daptomycin, gentamicin, levofloxacin, streptomycin	*aadE, tetM, tetS*	Obioha et al. (2023)
	Abattoir environment, slaughtered cattle, stool from abattoir workers	Extended spectrum beta lactamase *Escherichia coli*	Ampicillin, sulfonamides, azithromycin, cefotaxime, ceftazidime, chloramphenicol, gentamicin, nalidixic acid, tetracycline, trimethoprim	*aac, aad, Bla*_*CTX‐M*_, *cat, cml, dfr, erm, floR, fosA, lla, mcr, mdf, mef, mph, qacE, qepA, qnrS, sul, tet*	Aworh et al. (2022)
	Cow meat faeces and environmental sample	*Escherichia coli* O157:H7	Amoxicillin‐clavulanic acid, ampicillin, ceftazidime, cefuroxime, ciprofloxacin, gentamicin, nitrofurantoin	*—*	Ajuwon et al. (2021)
	Slaughterhouse workers faeces	*Escherichia coli*	Ampicillin, azithromycin, cefotaxime, ceftazidime, chloramphenicol, ciprofloxacin, gentamicin, nalidixic acid, sulfonamides, tetracycline, trimethoprim	*—*	Aworh et al. (2021)
Sierra Leone	Poultry faeces	*Escherichia coli*	Ampicillin, cefoxitin, chloramphenicol, erythromycin, penicillin, streptomycin, sulfafurazole, tetracycline	*—*	Mansaray et al. (2022)
South Africa	Broiler chickens at abattoir	*Proteus mirabilis*	Amikacin, amoxicillin‐clavulanic acid, aztreonam, cefazolin, cefepime, ciprofloxacin, ertapenem, gentamicin, imipenem, levofloxacin, meropenem, nalidixic acid, norfloxacin	*catI, catII, mcr‐1, mecA, qnrA, qnrD*	Ramatla et al. (2024)
	Pig manure and pine wood mixture (75:25)	*Campylobacter* species, *Escherichia coli, Yersinia* species	Amoxicillin, amoxicillin‐clavulanate, ampicillin, cefotaxime, chloramphenicol, ciprofloxacin, erythromycin, gentamicin, nalidixic acid, nitrofurantoin, streptomycin, sulfamethoxazole, tetracycline, trimethoprim‐sulfamethoxazole	*—*	Manyi‐Loh et al. (2023)
	Chicken litter	*Escherichia coli*	Amikacin, amoxicillin‐clavulanic acid, ampicillin, cefepime, cefolexin, cefotaxime, cefoxitin, ceftriaxone, chloramphenicol, ciprofloxacin, gentamicin, imipenem, nalidixic acid, tetracycline, tigecycline, trimethoprim‐sulfamethoxazole	*—*	Fatoba et al. (2022)
	Fruits and vegetables	*Listeria monocytogenes*	Amikacin, amoxicillin, ampicillin, ampicillin‐sulbactam, cefotetan, ceftriaxone, chloramphenicol, ciprofloxacin, clarithromycin, doripenem, ertapenem, erythromycin, fosfomycin, gentamicin, imipenem, oxytetracycline, penicillin G, streptomycin, sulfamethoxazole, trimethoprim, trimethoprim‐sulfamethoxazole, vancomycin	*—*	Kayode and Okoh (2022)
	Pig farm	*Enterococcus* species	Chloramphenicol, ciprofloxacin, erythromycin, gentamicin, nitrofurantoin, quinupristin‐dalfopristin, streptomycin, sulfamethoxazole‐trimethoprim, teicoplanin, tetracycline	*aac(6′)‐le‐aph(2″)‐la, aph(3′)‐IIIa, ermB, tetK, tetM*	Badul et al. (2021)
	Chicken litter	*Enterococcus* species	Ampicillin, ciprofloxacin, erythromycin, imipenem, levofloxacin, nitrofurantoin, quinupristin‐dalfopristin, streptomycin, sulfamethoxazole‐trimethoprim, tetracycline	*—*	Fatoba et al. (2021)
	Chickens	*Salmonella* species	Amoxicillin‐clavulanic acid, ampicillin, cefotaxime, ceftazidime, chloramphenicol, ciprofloxacin, doxycycline, erythromycin, gentamicin, kanamycin, nalidixic acid, norfloxacin, oxytetracycline, spectinomycin, streptomycin, sulfamethoxazole‐trimethoprim	*—*	Mokgophi et al. (2021)
Tunisia	Broiler chicken	*Salmonella* species	Amoxicillin, amoxicillin‐clavulanic acid, aztreanam, cefalotine, cefepime, cefotaxime, cefoxitin, ceftazidime, ceftriaxone, chloramphenicol, ciprofloxacin, colistin, enrofloxacin, ertapenem, florfenicol, gentamicin, nalidixic acid, streptomycin, sulfonamides, tetracycline, trimethoprim‐sulfamethoxazole	*bla*_*CTX‐M*_, *dfrA, tetA, tetB*	Oueslati et al. (2021)
Zambia	Raw dressed broilers	*Listeria monocytogenes*	Chloramphenicol, clindamycin, gentamicin, levofloxacin, penicillin G, tetracycline, trimethoprim‐sulfamethoxazole	*—*	Mpundu et al. (2022)
—	Meat and meat products	*Staphylococcus aureus*	Amikacin, ampicillin, cefoxitin, ceftaroline, ciprofloxacin, doxycycline, erythromycin, gentamicin, kanamycin, minocycline, oxacillin, penicillin G, tetracycline, vancomycin	*ant(4′), ant(4′)‐Ia, aph(3′)‐IIIa, blaZ, ermA, ermC, ermT, msrA, tetK, tetL, tetM*	(Thwala et al. (2021))

Animals may transmit antibiotic‐resistant bacteria to humans through their consumption as food. Resistant bacteria have been isolated from various food products, including milk, eggs, meat, and even vegetables (Ma et al. 2021). Contamination at the farm level can persist in raw and undercooked foods, posing a health risk to consumers (Samtiya et al. 2022). Farm bioaerosols generated during agricultural activities serve as reservoirs for antibiotic‐resistant bacteria which can be transmitted to humans through inhalation (Bai et al. 2022). However, the dispersal patterns and distances of airborne bacteria and resistance genes remain poorly understood (Bai et al. 2022).

The transmission of antibiotic‐resistant bacteria is a One Health issue, as resistance can be transmitted between humans, animals, and the environment (Velazquez‐Meza et al. 2022; Jin et al. 2023), facilitating the exchange of resistance genes. Humans may then acquire these bacteria through direct contact or indirectly via contaminated surfaces (fomites). As a result, the natural environment is recognized as a significant route for the transmission of antibiotic‐resistant bacteria to humans (Endale et al. 2023).

## 6 Strategies to Reduce Antibiotic Use in Livestock Production

The misuse of antibiotics in livestock farming significantly contributes to the development of antibiotic resistance in bacteria in animals and humans (Bukari et al. 2025; Thakar et al. 2025). Reducing antibiotic use in livestock can help minimize exposure of bacteria to antibiotics, thus lowering the incidence of resistance. Strategies to achieve this include adopting alternative feed additives for growth promotion, using alternative therapeutic agents for treating animal infections, educating farmers on the risks of antibiotic misuse, ensuring accurate reporting of antibiotic usage, and enforcing regulations to prevent misuse.

### 6.1 Alternative Growth Promoters/Feed Additives

Probiotics, prebiotics, vitamin C, phytogenics, and immune stimulants have been proven to be effective alternatives to antibiotics growth promoters (Shehata et al. 2022). Phytogenic animal feeds, also known as phyto‐biotics or plant‐based medicines, are plant‐derived compounds used in animal nutrition to enhance production. These additives require minimal processing and can effectively replace antibiotic growth boosters in feed (Rossi et al. 2020). Phytogenics support animal growth by improving food intake, modulating ruminal fermentation, enhancing nutrient digestion and absorption, and exerting anabolic effects on target tissues (Valenzuela‐Grijalva et al. 2017). They may also stimulate olfactory nerves and taste buds, regulate metabolism, and promote muscle development, thereby increasing feed consumption and weight gain (Valenzuela‐Grijalva et al. 2017). Probiotics help maintain gut microbial balance of bacteria through competitive exclusion and preventing overgrowth of harmful bacteria, while prebiotics support the proliferation of beneficial lactic acid bacteria, enhancing pathogen resistance and improving feed utilization for better growth (Shehata et al. 2022).

Various feed additives have been reported to enhance the productivity of pigs and poultry, including omega‐3 fatty acids, immunoglobulins, organic and inorganic acids, zinc oxide, yeast‐derived β‐glucans, essential oils, prebiotics, probiotics, threonine, cysteine, and herbal supplements (Ghimpețeanu et al. 2022). Herbs such as thyme, oregano, rosemary, marjoram, yarrow, garlic, ginger, green tea, black cumin, coriander, and cinnamon have been used as growth promoters in poultry (Lillehoj et al. 2018). Essential oils like thymol, carvacrol, cinnamaldehyde, eugenol, coriander, star anise, ginger, garlic, rosemary, turmeric, basil, caraway, lemon, and sage have shown similar benefits (Lillehoj et al. 2018). A herbal feed additive containing benzoic acid and essential oils (thymol, eugenol, and piperine) enhanced piglet growth by improving average daily gain and feed intake (Silva Júnior et al. 2020). Other antibiotic alternatives include xylanases, which improve broiler growth performance (Nusairat and Wang 2020), algae‐derived polysaccharides (ADP), which enhance antioxidant capacity and gut health in broilers (Liu et al. 2020), and mesobiliverdin IX alfa (MBV)‐enriched microalgae *Spirulina* extracts, which promote animal growth (Chang et al. 2021).

### 6.2 Alternative Strategies for the Prevention of Disease and the Treatment of Livestock

Reliance on the use of antibiotics can be reduced by using alternative treatments. In developing countries, medicinal plants are widely used in veterinary care, with more than 500 herbal remedies known to treat animals across 18 regions of southern Africa (Ivanova et al. 2024). Ethnomedical practices in rural areas involve using plants, which are either dried, crushed, or used raw as effective replacements for conventional antibiotics in treating infections. These plants are used to treat a variety of conditions such as anthrax, general fever, swollen glands, lung diseases, constipation, indigestion, cancer, and snakebite (Hu et al. 2019). Some herbal extracts, including tannins, saponins, and essential oils, are particularly valued for their potent natural antimicrobial, antioxidant, anti‐inflammatory, and immunomodulatory properties, making them strong alternatives to traditional antibiotics (Farha et al. 2020; Hernández‐González et al. 2021). Quercetin demonstrated antibacterial activity against *Salmonella enterica* serovar Typhimurium, *P. aeruginosa, E. coli,* and *S. aureus* in the chicken intestine (Iqbal et al. 2020). Dietary resveratrol has also been reported to inhibit *Salmonella* species and *E. coli*, which cause intestinal diseases in piglets (Wang et al. 2024).

The inclusion of oregano essential oils into chicken diets, together with *Saccharomyces cerevisiae* administration, has been proposed as an integrated strategy to prevent intestinal colonization by *Salmonella* (Lupia et al. 2024). Essential oils from *Cinnamomum verum* and *Syzygium aromaticum* have also demonstrated potential for disinfection, both individually and in combination (Ebani et al. 2019). Oregano essential oils exhibited inhibitory effect against *Candida* species, *Salmonella* species, *Listeria monocytogenes,* and *Streptococcus pyogenes* (Cui et al. 2024). Carvacrol inhibited *S. pyogenes* biofilms with a minimum biofilm inhibitory concentration (MBIC) of 125 μg/mL (Wijesundara et al. 2022).

Antimicrobial peptides are low‐molecular weight molecules, forming a diverse and abundant group of biomolecules produced in several animal and plant cells. These peptides exhibit biological activity against bacteria, viruses, and fungi, making them promising alternatives for treating animal infections and diseases. They possess multiple antimicrobial functions, acting on different cellular targets such as DNA, RNA, and regulatory enzymes. Additionally, they can kill multidrug‐resistant bacteria like methicillin‐resistant *S. aureus* (Silveira et al. 2021). Common antimicrobial peptides that have been reported for the treatment of bacterial infection in animals include plectasin, nisin, lactoferricin, indolicin, and cethelicidins (Saeed et al. 2022). Indolicin was used against *Campylobacter jejuni, E. coli, L. monocytogenes, P. aeruginosa, S. typhimurium, S. aureus, S. epidermidis,* and *S. agalactiae* in cattle (Valdez‐Miramontes et al. 2021). Colicins, a class of bacteriocins produced by *E. coli* have been used to treat diarrhoea caused by enterotoxigenic *E. coli* in piglets (Silveira et al. 2021). A case study reports ethnoveterinary use of plants in Dugda District, Oromia National Regional State, Ethiopia, revealing the use of 64 plants belonging to 58 genera and 33 families, with herbs being the most frequently employed (32.81%) (Oda et al. 2024).

### 6.3 Regulations Against the Misuse of Antibiotics in Livestock Production

Many developed countries have imposed restrictions on the use of antibiotics in animal production for growth promotion (Ma et al. 2021; Da Silva et al. 2023). The effectiveness of such bans is still debatable, as there have been recent reports indicating that antibiotics continue to be used as feed additives even in some developed countries (Aminullah et al. 2025). Namibia became the first African country to ban the use of antibiotics as growth promoters in its beef industry in 1991 (Haimbodi et al. 2024). While some African countries have enacted laws against the misuse of antibiotics in livestock, these regulations are often not strictly enforced, undermining their intended impact. Implementing and enforcing these laws is crucial to limit antibiotic use in healthy animals, preserve the effectiveness of antibiotics for human medicine and mitigate the growing threat of antibiotic‐resistant bacteria (Ducrot et al. 2021).

A review of antibiotic use across 31 African countries in 2021 revealed significant variation in the development and implementation of national antibiotic resistance plans (Mikecz et al. 2020). Countries like Angola and Guinea have no plans in place for antibiotic resistance, while several others, including Benin, Central African Republic, the Comoros, Lesotho, Mauritania, Rwanda and Togo, are still developing plans. Some countries, such as Ethiopia, Kenya, United Republic of Tanzania and Zambia, have fully implemented plans with funding and others like Burkina Faso, Cote d'Ivoire, Gabon, Democratic Republic of Congo, Mali, Malawi, Namibia, and Seychelles have established plans. Chad, Ghana, Liberia, Malawi, Mauritius, Mozambique, Nigeria, Sierra Leone, South Africa, Uganda, and Zimbabwe have action plans, operational plans with monitoring arrangements (Mikecz et al. 2020).

Only 17 out of the 31 countries have laws regulating antibiotic prescriptions and sales for animal use, viz., Angola, Benin, Burkina Faso, Central African Republic, Cote d'Ivoire, Ethiopia, Guinea, Kenya, Mali, Mauritius, Namibia, South Africa, Uganda, United Republic of Tanzania, Zambia, and Zimbabwe and just eight countries, viz., Angola, Cote d'Ivoire, Ethiopia, Kenya, Namibia, United Republic of Tanzania, Zambia, and Zimbabwe have laws against the use of antibiotics as animal growth promoters (Mikecz et al. 2020). The review highlights the need for more African nations to adopt and enforce stricter regulations to combat antibiotic misuse in livestock production, particularly the use of antibiotics as growth promoters (Mshana et al. 2021).

Antibiotic resistance in low‐middle‐income countries is driven by complex factors and requires a multi‐sectoral approach to control its spread in humans and animals. To combat antibiotic resistance, African governments must enact regulations to ensure the rational use of antibiotics in animals (Chowdhury et al. 2021). Effective policies must regulate the use of antibiotics while ensuring their availability alongside other veterinary drugs (Ducrot et al. 2021). Misuse can be mitigated by better structuring, regulating, and monitoring the flow of veterinary drugs, particularly in rural areas. The support of organizations like World Organization for Animal Health (WOAH) is crucial in regulating illegal drug sales and establishing integrated monitoring systems for antibiotic use and resistance trends in animals, food products, and the environment (Bordier et al. 2020). The implementation of the livestock portion of the National Action Plan on antimicrobial resistance should be based on representative evidence from the entire country's livestock sector (Mikecz et al. 2020).

The problem of antibiotic resistance cannot be resolved by one nation or industry acting alone. At the international, regional, and national levels, a thorough, extensive One Health strategy is required in the fields of human and animal health, food production, environmental management, water and sanitation, education, and trade (Shabangu et al. 2025). The Terrestrial Animal Health Code of the World Organization for Animal Health recommended that the legislation in each country should include a provision of an exhaustive definition of a veterinary product, including any exclusions, regulate the import, manufacture, trade, distribution, and use of veterinary products (Tcheou et al. 2024). Also, the standards of the Codex Alimentarius sets global maximum residue limits for purposes of international trade and exchange, and stakeholders may define their maximum residue limits and risk management recommendations relating to residues of veterinary drugs in foodstuffs to the extent that this is justified and does not constitute an obstacle to international trade (Tcheou et al. 2024).

### 6.4 Raising Awareness Among Livestock Farmers

Farmers and veterinarians must understand responsible farming practices, antibiotic resistance, and antibiotic stewardship to prevent the development of antibiotic‐resistant bacteria (Llanos‐Soto et al. 2021). Raising awareness about proper antibiotic use and antibiotic resistance is essential to reduce the burden of resistance. Many farmers in rural African areas lack knowledge about the proper use of antibiotics and the risks they pose to the animals, humans, and the environment. The responsible use of antibiotics by animal farmers is closely linked to factors such as knowledge, attitudes, education level, and farming experience (Omolo et al. 2024).

An assessment of cattle farmers in Rwanda revealed that only 52.6% had correct knowledge of antibiotic use and 52.8% followed correct practices regarding antibiotic resistance, with positive attitudes, correct knowledge, and practices linked to higher educational levels (Hirwa et al. 2024). Research in North‐western Ethiopia revealed inadequate knowledge and negative attitudes toward antibiotic use and resistance, as well as poor antibiotic use practices among animal farm workers, also related to education levels (Geta and Kibret 2021). In Togo, 21% of poultry farmers and 67% of pig farmers were unaware of antibiotics, while 39% and 57% did not know about antibiotic resistance, respectively. In fact, 19% and 64% did not have adequate knowledge concerning antibiotics and antibiotic resistance, respectively (Bedekelabou et al. 2022). In Kenya, 41.9% of farmers believed that antibiotics should be used as animal growth promoters (Omolo et al. 2024). While Rwanda reports an average percentage in the knowledge of the proper use of antibiotics, Togo reports a low percentage, which reveals the difference in the level of education on antibiotic use in each country. These findings highlight a knowledge gap among African farmers.

To promote responsible antibiotic use, education on the risks of indiscriminate antibiotic use and the associated harm is essential. Better antibiotic stewardship is expected with more education and awareness programs. A cluster‐randomized control trial study protocol was designed in Ghana on vaccination uptake, antimicrobial usage and farmers' wellbeing. The study protocol contained 46 clusters (23 intervention groups and 23 control groups), which were assessed over a period of 12 months. The aim was to reduce the disease burden, the need to use antimicrobials in livestock and inspire farmers' wellbeing, the success of which informed the strategies to tackle the impact of infectious livestock diseases on food security, public health and farmers' wellbeing (Nuvey et al. 2025).

Surveillance systems are also needed to track the use of antibiotics within Africa and inform decisions on control measures. Reporting and surveillance for estimating antimicrobial consumption is essential to set up measurable policy targets and assess the effectiveness of interventions and policies aimed at reducing the indiscriminate use of antimicrobials in animals (Ching et al. 2024). A mixed‐methods study was used to investigate the performance in addressing antimicrobial resistance in Kenya, Tanzania, Uganda, and Zambia. The study revealed significant gaps in tackling antibiotic resistance in sub‐Saharan Africa, including limited capacity in the animal, environmental, and agricultural sectors to conduct surveillance. There was also a lack of data on antibiotic resistance in the region, and this hindered the creation of a national database, making it difficult to effectively control the misuse of antibiotics (Matee et al. 2023).

Veterinarians play a crucial role in reducing antibiotic resistance by educating clients regarding the judicious use of antibiotics. Successful antibiotic resistance reduction programs often depend on changing the attitudes held by veterinarians and their clients toward antibiotic prescription and administration (Llanos‐Soto et al. 2021). Gaps in farmers' knowledge, attitudes, and practices can be addressed through awareness campaigns and training programs on antibiotic use, resistance, and stewardship (Mokhutsoane and Essack 2024). Understanding farmers' practices on antibiotic use, resistance, and stewardship is essential for developing effective strategies, regulations, and policies to promote prudent antibiotic use and prevent or limit the emergence of antibiotic resistance in food animal production systems (Farrell et al. 2021).

## 7 Conclusions

The persistent use of antibiotics for infection prevention, treatment and as feed additives for growth promotion significantly contributes to the growing burden of antibiotic resistance in Africa. Misuse of antibiotics in animal production leads to resistance in food animals, impacting animal health and potentially causing resistant infections in humans. Resistant bacteria can be transferred from animals to humans through various routes, resulting in infections that are difficult to treat, longer treatment durations, and increased healthcare costs. Given the environment's role in the spread of resistance, antibiotic resistance has become a One Health challenge, requiring a multi‐sectoral approach. To address this, antibiotic use in animal production must be minimized, with plant‐based alternatives such as prebiotics, probiotics, and phyto‐biotics being promoted for growth and disease treatment. Educating farmers and veterinarians on the proper use and potential harm of antibiotics is critical, as their awareness is key to the responsible administration of antibiotics. Governments must also implement and enforce regulations to prevent the misuse of antibiotics, particularly as growth promoters in agriculture.

## Author Contributions

**Mercy A. Alabi:** writing – original draft, conceptualization. **Hafizah Y. Chenia:** conceptualization, writing – review and editing; supervision. **Johnson Lin:** conceptualization, writing – review and editing, supervision.

## Conflicts of Interest

The authors declare no conflicts of interest.

## Data Availability

Data sharing not applicable to this article as no data sets were generated or analyzed during the current study.
